# Association of Plant-Based Protein Intake with Cognitive Function in Adults with CKD

**DOI:** 10.34067/KID.0000000000000278

**Published:** 2023-10-27

**Authors:** Luis Perez, Zhiying You, Jessica Kendrick

**Affiliations:** Division of Renal Diseases and Hypertension, University of Colorado Anschutz Medical Campus, Aurora, Colorado

**Keywords:** cognition, nutrition

## Abstract

**Key Points:**

- Higher plant protein intake was associated with higher cognitive scores in people with kidney disease.
- Future trials are needed to determine whether increasing plant protein intake improves measures of cognition in patients with kidney disease.

**Background:**

Patients with CKD have accelerated cardiovascular and cognitive aging when compared with the non-CKD population. This cognitive decline contributes to excessive rates of physical and functional decline, reduced quality of life, and mortality in the CKD population. Mediterranean diets, a plant-forward diet, have been associated with positive cognitive performance in the general non-CKD population and with some beneficial outcomes in CKD. However, it is still unclear whether plant-based diets are associated with cognitive decline in patients with CKD.

**Methods:**

Using the National Health and Nutrition Examination Survey 2011–2012 and 2013–14 data, we conducted a secondary analysis evaluating the relationship of plant-based and unprocessed plant protein with cognitive outcome measures in eligible participants aged 60 years and older. All data were extracted from the available National Health and Nutrition Examination Survey demographic, questionnaire, examination, and laboratory data. CKD was calculated and defined in participants as urine albumin to creatinine ratio ≥30 mg/g and/or eGFR <60 ml/min. In incremental models, we adjusted for total energy intake, age, sex, race/ethnicity, body mass index, total energy intake, diabetes, hypertension, education, smoking, and alcohol.

**Results:**

Higher plant-based protein above median dietary intakes was significantly associated with higher executive function scores in participants with CKD (*P* < 0.05). For all patients and in those with CKD, plant-based protein was significantly associated (*P* < 0.05) with higher composite cognitive scores in nearly all statistical models. Higher unprocessed plant protein was significantly associated (*P* < 0.05) with higher composite cognitive scores in all categorical models.

**Conclusions:**

Higher plant protein intake was a significant predictor of certain individual and composite cognitive score measures within the general and in the CKD population. Future interventional trials are needed to determine whether increasing plant-based protein intake improves measures of cognition in patients with CKD.

## Graphical abstract

**Figure:**
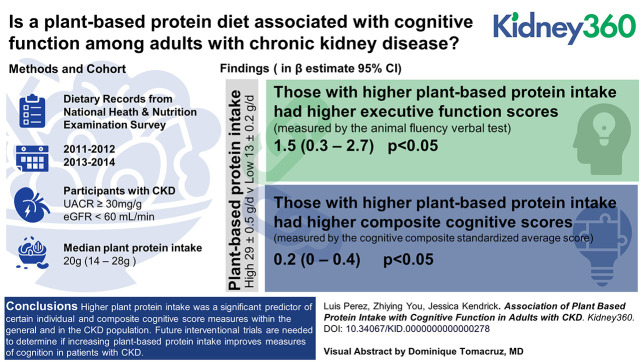

## Introduction

Patients with CKD have accelerated cardiovascular and cognitive aging when compared with non-CKD populations, which contributes to cognitive impairment. Furthermore, progression of kidney disease contributes to cognitive decline with estimates of 20%–50% of older adults with CKD (age 50 years or older) having cognitive impairment or dementia. This cognitive decline contributes to excessive rates of physical/functional decline, reduced quality of life, and mortality in the CKD population. It is of utmost importance to identify ways to reduce cognitive impairment in CKD.

In the general aging population, a Mediterranean diet has been associated with cognitive performance.^1–3^ Diet may be of crucial importance in cognitive impairment in CKD because the ability to excrete the dietary acid load is impaired leading to a positive acid balance. This acid retention contributes to increased inflammation and oxidative stress which may lead to vascular/cerebrovascular damage and cognitive decline. Dietary acid load in CKD is strongly linked to the source of protein consumed, with animal-based protein sources resulting in higher dietary acid load compared with plant-based protein sources. Plant-based diets are also associated with improved blood pressure in CKD compared with animal-based diets. Diets higher in plant-based proteins are associated with better survival in patients with CKD. However, it is still unclear whether plant-based diets are associated with cognitive decline in patients with CKD because there is a lack of research on this topic. The purpose of this study was to evaluate the association of predominately plant-based protein diets with cognitive function in adult participants with CKD. To provide a nuanced understanding of food quality, we used a modified NOVA classification scheme,^4^ focusing specifically on unprocessed or minimally processed plant-based foods, to allow us to consider the bioavailability of key nutrients, such as potassium and phosphorus, in the context of CKD.

## Methods

### Study Population and Design

Using the National Health and Nutrition Examination Survey (NHANES) 2011–2012 and 2013–14 datasets,^5^ we included eligible participants aged 60 years and older with cognitive and dietary data available. All data were extracted from the available NHANES demographic, questionnaire, examination, and laboratory data. CKD was calculated and defined in participants as urine albumin to creatinine ratio ≥30 mg/g and/or eGFR <60 ml/min. The 2021 Chronic Kidney Disease Epidemiology Collaboration eGFR equation^6^ was calculated for all included participants based on available serum creatine values. The laboratory collection protocol, procedures, and analyses have been previously described in the NHANES program.^5^ NHANES demographic survey data were originally collected by in-home training interviewers using Computer-Assisted Personal Interviewing system. Participant education levels for < ninth grade and 9–12th grade were collapsed into a “less than high school” category for covariate analysis.

Dietary records collected from NHANES were used for analysis of nutritional variables and outcomes. Trained interviewers originally conducted two 24-hour dietary recalls as part of the dietary interviews within NHANES, as is previously described.^5,7^ Study analyses included participants with complete and reliable 2-day 24-hour dietary records. We calculated protein intake type (*e.g.*, plant, animal, dairy, or mixed) from dietary records by linking the Food and Nutrient Database for Dietary Studies with food composition data provided by the United States Department of Agriculture Nutrient Database for Standard Reference.^7^ In general, this methodology separates protein intake classifications into animal, plant, dairy, and mixed protein categories.^7^ Animal protein includes foods such as, but not limited to, chicken and beef while plant protein includes foods such as grains, nuts, fruits, and vegetables.^7^ We excluded dairy (*e.g.*, milk, yogurt, ice cream) and mixed proteins (*e.g.*, pizza, burgers, mixed dishes) for the purposes of these analyses.

To address food and diet quality in the context of CKD, we used a modified NOVA^4^ classification scheme that also considers the bioavailability of key nutrients, such as potassium and phosphorus. To summarize, NOVA categorizes foods into four category groups: (*1*) unprocessed or minimally processed foods, (*2*) processed culinary ingredients, (*3*) processed foods, and (*4*) ultraprocessed foods.^4^ Because this study was focused on the potential health benefits of unprocessed or minimally processed plant-based foods (*i.e.*, generally positive eating habits when compared to more processed or undesirable foods), our analyses specifically targeted foods in NOVA's group 1 that are also plant-based protein sources (*i.e.*, unprocessed plant protein intake and *e.g.*, fruits, vegetables, legumes, seeds). This approach allows us to explore the health outcomes associated with foods that are not only minimally processed but also low in bioavailable potassium and phosphorus, which is particularly relevant for patients with CKD. Plant protein intake was examined categorically with high (mean 30.5±0.3 g/d) versus low (mean 13.5±0.1 g/d) defined by median levels (20.0 [14.1–27.7] g/d) in all included participants. A similar approach was used with unprocessed plant protein intakes, which had a median intake of 4.5 (2.1–8.8) g/d.

Cognitive outcomes from the study were conducted cross-sectionally by NHANES in household interviews or within Mobile Examination Centers in participants aged 60 years and older.^5^ The primary cognitive outcome measures included (*1*) Consortium to Establish a Registry for Alzheimer's disease (CERAD) Word Learning subtest^8^; (*2*) the Animal Fluency verbal fluency test (AFT)^9^; and (*3*) the Digit Symbol Substitution test (DSST).^10^ This CERAD subtest consists of three consecutive learning trials, a delayed recall, and focuses on immediate, delayed memory and learning ability for new verbal information.^11^ For the CERAD test, the maximum individual test score was ten, and a total score was calculated by summing all three trials similarly to previous studies.^12,13^ The AFT evaluates executive function, and the DSST evaluates processing speed, attention, and working memory. All cognitive outcome measures (CERAD, AFT, and DSST) were merged into raw composite scores.^12,13^ In addition, standardized (z-scores) were generated for each of the three outcome measures and combined into cognitive composite standardized scores (sum of z-scores) and cognitive composite standardized average scores (average of z-scores) for analyses.

### Statistical Analyses

Descriptive statistics, from participants with complete and weighted results, were used to summarize all data. For example, mean±SEM or median and interquartile range were calculated for a continuous variable if appropriate, and frequency and proportion were reported for a categorical or ordinal variable. Data transformation was performed before further analysis if appropriate. Linear regression analysis was performed to examine the association between plant-based protein intake and cognition. Plant-based protein, unprocessed plant protein, and animal protein were analyzed as continuous variables and as categorical variables cut at the median intake value (*i.e.*, high versus low). Unadjusted statistical models included only plant protein variables as a predictor in the CKD-only participant group and both plant protein variables and eGFR in all eligible participants group. Adjusted model one (model 1) included the unadjusted model and total energy intake,^14^ age, sex, race/ethnicity, and body mass index as covariates. The second model (model 2) included model 1 and diabetes, hypertension, education, smoking, and alcohol. All statistical analyses included the appropriate NHANES data weights. In the NHANES raw data, a set of adjusted weights, WTDR2D, is to be used only when an analysis uses both day 1 and day 2 dietary data, which is the case of the current analysis. Because combined data of 4 years were used in this study, the weights for 4 years calculated as WTDR2D4=WTDR2D/2 was used in analysis. The two-sided significance level of 0.05 was used in making a conclusion. All analyses were performed using SAS software, version 9.4 (SAS Institute Inc.).

## Results

### Study Participants

We included 2176 total NHANES participants, of whom 679 participants had CKD and complete data available. Demographics for all participants and those with CKD are presented in Table 1. The mean age and eGFR of all eligible included participants were 69.0±0.3 years and 77.3±0.5 ml/min per 1.73 m^2^, respectively. More than half of included participants were male (51.4%), and the majority were Non-Hispanic White (51.5%). Individuals in the CKD group were older (Table 1) and generally had numerically lower cognitive test scores than those without CKD (Table 2). In addition, total protein intake was numerically lower in individuals with CKD compared with those without CKD.

**Table 1:** Included participant demographics and characteristics from National Health and Nutrition Examination Survey, 2011–12 and 2013–14

Characteristic	Values	Values	Values
	All Participants (*n*=2176)	Non-CKD Only (*n*=1497)	CKD Only (*n*=679)
Age, yr	69.0±0.3	67.9±0.3	71.9±0.5
BMI, kg/m^2^	29.0±0.3	28.8±0.4	29.6±0.3
Albumin creatinine ratio, mg/g (natural log)	2.1 (1.7–2.9)	2.0 (1.6–2.4)	3.5 (2.2–4.1)
eGFR, ml/min	77.3±0.5	83.2±0.5	61.0±1.4
**Sex, *No.* (%)**			
Male	1058 (47.1%)	725 (48.4%)	333 (49.0%)
Female	1118 (52.9%)	772 (51.6%)	346 (51.0%)
**Race, *No.* (%)**			
Non-Hispanic Asian	158 (4.1%)	123 (8.2%)	35 (5.2%)
Non-Hispanic Black	475 (7.6%)	298 (19.9%)	177 (26.1%)
Mexican American	187 (3.3%)	143 (9.6%)	44 (6.5%)
Other Hispanic	211 (3.9%)	157 (10.5%)	54 (8.0%)
Other race—including multiracial	24 (1.1%)	16 (1.1%)	8 (1.2%)
Non-Hispanic White	1121 (80.0%)	760 (50.8%)	361 (53.2%)
**Education completed, *No.* (%)**			
Less than high school	507 (14.8%)	310 (20.7%)	197 (29.0%)
High school	525 (22.3%)	353 (23.6%)	172 (25.3%)
Some college or associates	617 (29.7%)	435 (29.1%)	182 (26.8%)
College graduate or above	527 (33.2%)	399 (26.7%)	128 (18.9%)
Diabetes, *No.* (%)	519 (18.1%)	268 (17.9%)	251 (37.0%)
Hypertension, *No.* (%)	1338 (57.6%)	830 (55.4%)	508 (74.8%)
**Protein intake, g/d**			
Total protein intake	75.3±1.0	77.3±1.1	70.0±1.4
Animal protein intake	22.6±0.4	22.6±0.6	22.5±0.8
Plant protein intake	22.9±0.2	23.7±0.5	20.7±0.6
Dairy protein intake	8.8±0.2	10.7±0.4	9.1±0.4
Mixed protein intake	18.0±0.4	19.3±0.9	17.1±1.4
Unprocessed plant protein intake	6.3±0.2	6.6±0.3	5.5±0.3

All data are presented as mean±SEM or median (IQR). Percent values are rounded to one decimal place. Values reported from participants with complete data are used in weighted analyses. BMI, body mass index; IQR, interquartile range.

**Table 2:** Variables and outcomes across low versus high plant protein intake and CKD status

Protein Group, *No.*	All Participants		Non-CKD		CKD	
	Low Plant Protein Intake, *N*=1081	High Plant Protein Intake, *N*=1095	Low Plant Protein Intake, *n*=681	High Plant Protein Intake, *n*=816	Low Plant Protein Intake, *n*=400	High Plant Protein Intake, *n*=279
**Variable**						
Age, yr	69.5±0.2	69.3±0.2	68.2±0.2	68.2±0.2	71.8±0.4	72.3±0.4
BMI, kg/m^2^	29.2±0.2	28.8±0.2	29.2±0.2	28.6±0.2	29.8±0.3	29.3±0.4
Albumin creatinine ratio, mg/g	10.3 (6.0–25.2)	8.9 (5.5–18.3)	7.9 (5.4–12.3)	7.4 (5.1–11.7)	37.3 (10.7–88.2)	39.5 (11.1–86.7)
eGFR, ml/min	74.2±0.6	77.8±0.5	83.2±0.5	83.3±0.4	59.0±1.0	61.6±1.3
Protein intake						
Total protein intake, g/d	61.4±0.8	83.4±0.9	63.8±1.0	84.4±1.0	57.5±1.2	81.2±28.3
Animal protein intake, g/d	21.1±0.6	24.7±0.6	21.4±0.7	24.3±0.7	20.5±0.9	25.9±20.8
Plant protein intake, g/d	13.5±0.1	30.5±0.3	13.8±0.2	30.9±0.4	13.0±0.2	29.5±8.9
Unprocessed plant protein intake, g/d	3.7±0.1	9.6±0.3	4.0±0.1	9.7±0.3	3.2±0.1	9.2±8.2
**Cognitive scores**						
CERAD total score	18.8±0.1	19.5±0.1	19.3±0.2	20.0±0.2	17.9±0.3	18.3±0.3
Animal fluency test score	16.3±0.2	17.7±0.2	17.0±0.2	18.1±0.2	14.9±0.2	16.5±0.3
Digit symbol score	45.1±0.5	49.5±0.5	48.2±0.7	51.7±0.6	39.9±0.8	43.1±1.0
Composite raw score	80.2±0.7	86.7±0.7	84.6±0.9	89.7±0.7	72.7±1.1	77.9±1.3
Composite standardized score	−0.04±0.07	0.62±0.07	0.39±0.09	0.91±0.07	−0.77±0.11	−0.21±0.14
Composite standardized average score	−0.01±0.02	0.21±0.02	0.13±0.03	0.30±0.02	−0.26±0.04	−0.07±0.05

All data are presented as mean±SEM or median (IQR). Composite raw scores are the sum of individual raw values for the Consortium to Establish a Registry for Alzheimer disease, Animal Fluency Test, and Digit Symbol Score. Composite standardized scores are the sum of z-score for the three tests while composite standardized average scores are the average of these z-scores. BMI, body mass index; CERAD, Consortium to Establish a Registry for Alzheimer disease; IQR, interquartile range.

### Nutrient Intakes

Nutrient intakes across median (high versus low) plant protein intake groups and CKD status are presented in Table 2. The average plant protein intake was 22.9±0.2 g for all participants versus 20.7±0.6 g in the CKD group (Table 1). The overall median plant protein intake was 20.0 g (14.1–27.7 g) per day, which was used as a cutoff for defining high versus low levels of intake among all participants. The plant protein intake in the low and high groups was 13.5±0.1 and 30.5±0.3 g/d, respectively, among all participants (Table 2). In individuals with CKD, the low and high plant protein intake was 13.0±0.2 and 29.3±0.5 g/d, respectively. The overall median unprocessed plant protein intake was 4.5 g (2.1–8.8 g) per day among all participants. Dairy and mixed protein intakes are also reported in Table 1.

### Cognitive Outcomes

Cognitive score values in this study are reported in Table 2. When plant protein intake was examined continuously, in unadjusted models, there were significant associations (*P* < 0.05) among plant protein intake with individual CERAD, AFT, and DSST scores in all participants and those with CKD, except for the fully adjusted CERAD (*P* = 0.10) and DSST models (*P* = 0.09, Table 3). When examining plant protein intake categorically with individual cognitive scores, higher plant protein intake was associated with significantly higher CERAD, AFT, and DSST scores (*P* < 0.05), except after full adjustment for CERAD (*P* = 0.12) and DSST scores (*P* = 0.09) in all participants (Table 3). Only AFT scores were significantly associated with higher plant protein intakes (*P* < 0.05, Table 3) in patients with CKD.

**Table 3:** Association of plant-based protein intake with individual cognitive scores

Protein Group	CERAD Total Score	Animal Fluency Test Score		Digit Symbol Score		*P* Value
	*β*-Estimate (95% CI)	*P* Value	*β*-Estimate (95% CI)	*P* Value	*β*-Estimate (95% CI)	
**Plant protein, g (continuous)**						
All participants						
*Unadjusted*	0.02 (0.00 to 0.04)	<0.05	0.07 (0.04 to 0.10)	0.05	0.12 (0.04 to 0.20)	<0.01
*Model 1–2*	0.03 (0.00 to 0.05)	<0.05	0.06 (0.02 to 0.10)	<0.01	0.12 (0.03 to 0.21)	<0.05
*Model 2–2*	0.02 (−0.00 to 0.05)	0.10	0.05 (0.01 to 0.09)	<0.05	0.08 (−0.01 to 0.17)	0.09
CKD only						
*Unadjusted*	0.05 (0.00 to 0.10)	<0.05	0.07 (0.00 to 0.13)	<0.05	0.22 (0.04 to 0.39)	<0.05
*Model 1–2*	0.06 (0.00 to 0.12)	<0.05	0.07 (−0.03 to 0.16)	0.18	0.21 (−0.01 to 0.43)	0.06
*Model 2–2*	0.05 (−0.00 to 0.11)	0.06	0.06 (−0.03 to 0.15)	0.18	0.17 (−0.05 to 0.39)	0.12
**High plant protein versus low, g**						
All participants						
*Unadjusted*	0.56 (−0.07 to 1.21)	0.08	1.22 (0.46 to 1.97)	<0.01	2.92 (0.83 to 5.00)	<0.01
*Model 1–2*	0.68 (0.05 to 1.31)	<0.05	0.92 (0.25 to 1.59)	<0.01	2.88 (0.71 to 5.04)	<0.05
*Model 2–2*	0.49 (−0.14 to 1.12)	0.12	0.65 (0.01 to 1.30)	<0.05	1.81 (−0.30 to 3.91)	0.09
CKD only						
*Unadjusted*	0.93 (−0.47 to 2.33)	0.19	1.46 (0.10 to 2.82)	<0.05	4.72 (−0.30 to 9.74)	0.06
*Model 1–2*	0.86 (−0.41 to 2.13)	0.18	1.38 (−0.01 to 2.77)	0.05	3.99 (−0.80 to 8.78)	0.10
*Model 2–2*	0.83 (−0.37 to 2.04)	0.17	1.50 (0.29 to 2.70)	<0.05	3.67 (−0.49 to 7.82)	0.08

Unadjusted model: Plant protein+eGFR (all participants) or plant protein only (CKD only).

Model 1: Unadjusted model+total caloric intake+age+sex+race/ethnicity+BMI.

Model 2: Model 1+total caloric intake+diabetes+hypertension+education+smoking+alcohol.

Values are rounded to two decimal places. BMI, body mass index; CI, confidence interval; CERAD, Consortium to Establish a Registry for Alzheimer disease.

When analyzing plant protein continuously and across composite cognitive scores, higher plant protein intake was associated with higher cognitive composite scores in all models for all participants in those with CKD (all *P* < 0.05, Table 4). For unprocessed plant protein intake, there was generally no significant association with individual continuous cognitive scores, except for CERAD and AFT scores in all patients (Supplemental Table 1). However, categorical (high versus low) unprocessed plant protein intake was significantly associated with all individual cognitive scores except for CERAD scores in all patients and DSST scores in fully adjusted models (Supplemental Table 1). Unprocessed protein intake followed a similar pattern with composite cognitive scores in continuous models (Supplemental Table 2). However, unprocessed protein intake was significantly associated with all composite cognitive scores in all categorical models (Supplemental Table 2).

**Table 4:** Association of plant-based protein intake with composite cognitive scores

Protein Group	Cognitive Composite Raw Score		Cognitive Composite Standardized Score		Cognitive Composite Standardized Average Score	
	*β*-Estimate (95% CI)	*P* Value	*β*-Estimate (95% CI)	*P* Value	*β*-Estimate (95% CI)	*P* Value
**Plant protein, g (continuous)**						
All participants						
*Unadjusted*	0.21 (0.10 to 0.33)	<0.01	0.02 (0.01 to 0.04)	<0.01	0.01 (0.00 to 0.01)	<0.01
*Model 1–2*	0.21 (0.08 to 0.34)	<0.01	0.02 (0.01 to 0.04)	<0.01	0.01 (0.00 to 0.01)	<0.01
*Model 2–2*	0.15 (0.02 to 0.27)	<0.05	0.02 (0.00 to 0.03)	<0.01	0.01 (0.00 to 0.01)	<0.01
CKD only						
*Unadjusted*	0.34 (0.01 to 0.57)	<0.01	0.04 (0.01 to 0.06)	<0.01	0.01 (0.00 to 0.02)	<0.01
*Model 1–2*	0.34 (0.06 to 0.61)	<0.05	0.04 (0.01 to 0.07)	<0.05	0.01 (0.00 to 0.02)	<0.05
*Model 2–2*	0.29 (0.01 to 0.56)	<0.05	0.03 (0.00 to 0.06)	<0.05	0.01 (0.00 to 0.02)	<0.05
**High plant protein versus low, g**						
All participants						
*Unadjusted*	4.70 (1.64 to 7.76)	<0.01	0.51 (0.17 to 0.84)	<0.01	0.17 (0.06 to 0.28)	<0.01
*Model 1–2*	4.47 (1.55 to 7.40)	<0.01	0.47 (0.18 to 0.77)	<0.01	0.16 (0.06 to 0.26)	<0.01
*Model 2–2*	2.95 (0.13 to 5.78)	<0.05	0.32 (0.04 to 0.61)	<0.05	0.11 (0.01 to 0.20)	<0.05
CKD only						
*Unadjusted*	7.10 (−0.01 to 14.21)	0.05	0.73 (0.11 to 1.44)	<0.05	0.24 (0.00 to 0.48)	<0.05
*Model 1–2*	6.23 (−0.25 to 12.71)	0.06	0.66 (0.02 to 1.29)	<0.05	0.22 (0.01 to 0.43)	<0.05
*Model 2–2*	6.00 (0.36 to 11.63)	<0.05	0.65 (0.10 to 1.21)	<0.05	0.22 (0.03 to 0.40)	<0.05

Unadjusted model: Plant protein+eGFR (all participants) or plant protein only (CKD only).

Model 1: Unadjusted model+total caloric intake+age+sex+race/ethnicity+BMI.

Model 2: Model 1+total caloric intake+diabetes+hypertension+education+smoking+alcohol.

Values are rounded to two decimal places. Composite raw scores are the sum of individual raw values for the Consortium to Establish a Registry for Alzheimer disease, Animal Fluency Test, and Digit Symbol Score. Composite standardized scores are the sum of z-score for the three tests while composite standardized average scores are the average of these z-scores. BMI, body mass index; CI, confidence interval.

## Discussion

This study evaluated the association between plant-based protein intake and cognitive function in adults with and without CKD. Higher plant protein intake was a significant predictor of certain individual and composite cognitive score measures within the general and CKD population. Participants with CKD and high plant protein intake had significantly higher scores in certain memory and learning ability (CERAD score) and executive function (AFT score) models compared with those with low plant protein intake. Specifically, higher plant protein was most significantly associated with CERAD scores in continuous models and with AFT scores in the categorical models in CKD. Higher plant protein was significantly associated with nearly all composite cognitive scores in all models. In categorical models, higher unprocessed plant protein was significantly with individual and composite cognitive scores in nearly all models as well. To our knowledge, this is the first study examining the association of plant-based protein, as well as quality of protein, with cognitive function in participants with CKD.

Notably, the strongest associations were typically identified when plant-based protein was categorized into dichotomous high versus low levels at the median intake. The mean difference in plant protein intake between the low and high groups analyzed was approximately 16–17 g/d (Table 2). Plant protein accounts for a large proportion of the gap in total protein intake between the high/low plant protein groups at this threshold value, though dairy and mixed proteins likely also contribute to this. Animal protein intake was similar, generally within approximately 5 g/d (Table 2), across low or high plant protein intake groups. For some of the continuous models, 1 g of plant protein may be clinically inconsequential, but moving from low to high protein intake represents a significant increase in actual plant-based food consumption. One plant-based dietary intervention increased plant protein in the diet by approximately 19 g/d as part of a weight loss and insulin resistance intervention in overweight individuals.^15^ The observed difference in AFT and CERAD total scores was approximately 1–2 points between low and high protein intake groups (Table 2). The observed differences in composite standardized average (z) scores were approximately 0.20, which corresponds to an approximately eight percentile difference in standardized scores.

AFT scores had the most significant individual cognitive outcome associations with plant-based protein, which was followed by the CERAD test scores in participants with CKD. This indicates that dietary plant protein in this study was most closely associated with executive function (AFT) and memory (CERAD). Although the AFT and CERAD focus on different cognitive domains, both have been used in epidemiologic studies in diverse populations to elucidate normal, mild, and sever cognitive impairment.^5^ Studies have previously linked a healthy diet to cognitive and executive function, especially in adolescent populations.^16^ Dietary Approach to Stop Hypertension and Mediterranean diets may also slow cognitive decline,^17^ but this study focus on protein type, quantity, and quality is novel in CKD. Several of the plant-based protein intake models were no longer significant after full adjustment, potentially indicating that demographic factors (such as age, sex, race/ethnicity, diabetes, and education) explain the observed variation in cognitive scores. This was mostly seen within individual cognitive score models, such as DSST scores. The significance of many of the unprocessed plant protein models also potentially indicates that many of these associations may be mediated or driven by in part by whole, unprocessed plant foods. In addition, the mean unprocessed plant protein intakes typically ranged only from 3 to 10 g/d across groups. Our observations of a better relationship of high versus low protein intake with cognitive measures may indicate a threshold for plant-based protein intakes associated with improved cognitive performance.

Previous studies have investigated cognitive function in patients with CKD with mixed results. Previous studies have estimated that approximately 48% of individuals with CKD have moderate-to-severe cognitive impairment.^16^ Hailpern *et al.*^18^ previously found impaired cognitive function in the CKD population of NHANES III. Specifically, moderate CKD was associated with poorer learning, concentration, and visual attention but not with participant reaction times.^17^ Jovanovich *et al.*^19^ evaluated the association of markers of mineral metabolism with cognitive function in individuals with CKD. There was no association of vitamin D, parathyroid hormone, or fibroblast growth factor-23 with composite cognitive outcomes.^19^ In 2010, Tsai *et al.*^20^ cross-sectionally investigated markers of cognitive function in women with CKD. Tsai *et al.*^20^ did not focus on diet, but reported similar AFT scores as our current study (approximately 14–16 points) with no differences in AFT scores between healthy individuals and those with CKD, whereas we did when evaluating primarily across plant-based protein. In comparison, Martini *et al.*^21^ found also no differences in AFT test scores between patients with CKD and healthy controls but found that measures of memory were lower than controls. None of these previous studies examined the relationship between diet or plant-based protein intake and cognition in CKD.

Patients with CKD have reduced ability to excrete daily dietary acid load and may be in positive acid balance, although may not be clinically or overtly acidotic. Metabolic acidosis is associated with outcomes such as vascular dysfunction, inflammation, oxidative stress, as well as CKD progression and mortality.^22–24^ All these impairments of cardiovascular and cerebrovascular function may contribute to cognitive impairment and decline. In previous work, metabolic acidosis has been associated with cognitive impairment in 2853 older hypertensive participants with and without CKD.^25^ Currently, we have ongoing studies (NCT02915601 and NCT04600323) to evaluate the association of metabolic acidosis with cognitive impairment. These ongoing studies will evaluate whether chronic daily oral alkali therapy may reduce metabolic acidosis and/or improve cognitive performance. Conversely, it is possible that plant-based protein intake represents lower dietary acid load, higher dietary antioxidants, or other healthy habits that may improve cognitive performance or be associated with increased cognitive function. Although oral alkali therapy may improve metabolic acidosis in CKD, several studies have demonstrated that base-producing fruit and vegetables may have similar to or potentially greater effects at doing so.^26–29^ The base-producing effect of frequent or voluminous fruit and vegetables consumption may also have the potential benefit of reducing medication burden and symptoms while improving nutritional status and food security.^30^ Conversely, high sources of animal proteins may contribute to positive dietary acid intake.

Our current study was limited by the narrow band of laboratory carbon dioxide values and the lack of severe metabolic acidosis or advanced CKD in the NHANES population. However, our study is novel in evaluating dietary intake and measures of cognitive function in a relatively large sample size. We also did not adjust for other factors such as medications and socioeconomic status. However, we did adjust for major factors such as age, sex, race/ethnicity, education, diabetes, hypertension, education, smoking, and alcohol use. In addition, we acknowledge the limitations of using the NOVA classification system, particularly the potential for misclassification and bias when focusing solely on nonmixed animal and plant protein sources. However, these sources did not dominate total protein intake, and the NOVA framework provides a useful starting point for exploring the health implications of food processing levels in the context of CKD. We acknowledge that eating habits are a marker of culture, and with our sample size, we were unable to examine this in more detail because of limitations in the NHANES dataset.

Future studies examining the effect of diet on cognitive outcomes should include biomarkers, dietary records, and examination of culture and socioeconomic status. We also aimed to elucidate the difference in quality or processing in plant-based protein, by focusing on the level of food/protein processing.^4,7^ More work is needed to systematically evaluate diet quality among mixed protein sources and processed foods. Increased fruit, vegetable, nut, and whole grain intake is a general health recommendation that is associated with many improved health outcomes, especially in CKD.^26–29,31^ It is clinically important to further the link between plant-based food consumption and independent or dependent measures of cognitive performance. Additional mechanisms for increased cognitive performance could be through reductions in blood pressure, decreased glomerular hyperfiltration, or other factors associated with increased plant-based consumption and decreased animal protein intake.^32,33^ Improved dietary patterns also decreases cardiovascular risk, another factor strongly associated with cognitive function. Future interventional trials are needed to determine whether increasing plant-based protein and protein quality intake improves cognition in patients with CKD.

## Supplementary Material

## Data Availability

Previously published data were used for this study. NHANES 2011–2012 and 2013–2014 data. These data are freely available.

## Supplemental Material

Supplemental Table 1. Association of unprocessed plant protein with individual cognitive scores.

Supplemental Table 2. Association of unprocessed plant protein with composite cognitive scores.
