# Supplementary material for: Association of Plant-Based Protein Intake with Cognitive Function in Adults with CKD
Source: Kidney360. 2023 Oct 27;4(11):1554–61. doi: 10.34067/KID.0000000000000278 (PMC10695646; doi:10.34067/KID.0000000000000278)
Supplement: Supplementary file 1 [file kidney360-4-1554-s001.pdf]

Supplementary Table 1. Association of Unprocessed Plant Protein with Individual Cognitive Scores

	CERAD total score		Animal fluency test score		Digit symbol score	
	β -estimate (95%CI)	P-value	β-estimate (95%CI)	P-value	β-estimate (95%CI)	P-value
**Unprocessed plant protein, g (continuous)**						
**All participants**						
Unadjusted	0.04 (0.00 to 0.07)	<0.05	0.05 (0.00 to 0.10)	<0.05	0.08 (-0.05 to 0.21)	0.20
Model 1	0.03 (0.00 to 0.07)	<0.05	0.05 (-0.01 to 0.10)	0.07	0.09 (-0.06 to 0.24)	0.22
Model 2	0.02 (-0.01 to 0.06)	0.18	0.03 (-0.02 to 0.07)	0.30	0.02 (-0.11 to 0.14)	0.79
**CKD only**						
Unadjusted	0.02 (-0.06 to 0.09)	0.64	0.02 (-0.06 to 0.10)	0.61	0.02 (-0.26 to 0.29)	0.91
Model 1	0.02 (-0.06 to 0.10)	0.63	0.06 (-0.03 to 0.15)	0.21	0.09 (-0.21 to 0.39)	0.54
Model 2	0.00 (-0.07 to 0.08)	0.90	0.04 (-0.04 to 0.12)	0.32	0.02 (-0.21 to 0.25)	0.86
**Unprocessed plant protein high vs. low, g**						
**All participants**						
Unadjusted	1.10 (0.44 to 1.68)	<0.05	1.11 (0.49 to 1.73)	<0.01	2.87 (0.84 to 4.90)	<0.01
Model 1	0.99 (0.37 to 1.60)	<0.01	1.07 (0.45 to 1.70)	<0.01	2.61 (0.77 to 4.46)	<0.01
Model 2	0.81 (0.14 to 1.49)	<0.05	0.76 (0.11 to 1.41)	<0.05	1.55 (-0.24 to 3.34)	0.09
**CKD only**						
Unadjusted	1.51 (0.17 to 2.85)	<0.05	1.43 (0.04 to 2.81)	<0.05	5.64 (0.36 to 10.92)	<0.05
Model 1	1.37 (0.12 to 2.62)	<0.05	1.59 (0.25 to 2.93)	<0.05	5.60 (1.16 to 10.05)	<0.05
Model 2	1.01 (-0.10 to 2.12)	0.07	1.14 (-0.01 to 2.29)	0.05	3.65 (-0.38 to 7.69)	0.07

Unadjusted Model: plant protein + eGFR (all participants) or plant protein only (CKD only)
Model 1: Unadjusted model + dietary animal protein + age + sex + race/ethnicity + BMI
Model 2: Model 1 + dietary animal protein + diabetes + hypertension + education + smoking + alcohol
Values are rounded to two decimal places. CERAD, Consortium to Establish a Registry for Alzheimer's disease; CKD, chronic kidney disease; BMI, body mass index; eGFR, estimated glomerular filtration.

Supplementary Table 2. Association of Unprocessed Plant Protein with Composite Cognitive Scores

	**Cognitive composite raw score**		**Cognitive composite standardized score**		**Cognitive composite standardized average score**	
	β -estimate (95%CI)	P-value	β-estimate (95%CI)	P-value	β-estimate (95%CI)	P-value
**Plant protein, g (continuous)**						
**All participants**						
Unadjusted	0.17 (-0.01 to 0.34)	0.06	0.02 (0.00 to 0.04)	<0.05	0.01 (0.00 to 0.01)	<0.05
Model 1	0.18 (-0.03 to 0.38)	0.09	0.02 (0.00 to 0.04)	<0.05	0.01 (0.00 to 0.01)	<0.05
Model 2	0.06 (-0.11 to 0.24)	0.46	0.01 (-0.01 to 0.03)	0.26	0.00 (-0.00 to 0.01)	0.26
**CKD only**						
Unadjusted	0.05 (-0.33 to 0.44)	0.78	0.01 (-0.03 to 0.05)	0.68	0.00 (-0.01 to 0.02)	0.68
Model 1	0.17 (-0.26 to 0.60)	0.43	0.02 (-0.03 to 0.06)	0.38	0.01 (-0.01 to 0.02)	0.38
Model 2	0.06 (-0.27 to 0.40)	0.70	0.01 (-0.03 to 0.04)	0.60	0.00 (-0.01 to 0.01)	0.60
**High plant protein vs. low, g**						
**All participants**						
Unadjusted	5.04 (2.50 to 7.57)	<0.01	0.59 (0.31 to 0.86)	<0.01	0.20 (0.10 to 0.29)	<0.01
Model 1	4.68 (2.31 to 7.04)	<0.01	0.55 (0.28 to 0.82)	<0.01	0.18 (0.09 to 0.27)	<0.01
Model 2	3.12 (0.65 to 5.59)	<0.05	0.40 (0.11 to 0.68)	<0.01	0.13 (0.04 to 0.23)	<0.01
**CKD only**						
Unadjusted	8.58 (1.44 to 15.71)	<0.05	0.90 (0.20 to 1.59)	<0.05	0.30 (0.07 to 0.53)	<0.05
Model 1	8.57 (2.60 to 14.53)	<0.01	0.89 (0.29 to 1.50)	<0.01	0.30 (0.10 to 0.50)	<0.01
Model 2	5.81 (0.52 to 11.09)	<0.05	0.63 (0.10 to 1.15)	<0.05	0.21 (0.03 to 0.38)	<0.05

Unadjusted Model: plant protein + eGFR (all participants) or plant protein only (CKD only)
Model 1: Unadjusted model + dietary animal protein + age + sex + race/ethnicity + BMI
Model 2: Model 1 + dietary animal protein + diabetes + hypertension + education + smoking + alcohol
Values are rounded to two decimal places. Composite raw scores are the sum of individual raw values for the Consortium to Establish a Registry for Alzheimer’s disease, Animal Fluency Test, and Digit Symbol Score. Composite standardized scores are the sum of z-score for the three tests, while composite standardized average scores are the average of these z-scores. CKD, chronic kidney disease; BMI, body mass index; eGFR, estimated glomerular filtration.
